# Relation of GRACE Risk Score to Coronary Lipid Core Plaques in Patients with Acute Coronary Syndrome

**DOI:** 10.3390/life13030630

**Published:** 2023-02-24

**Authors:** Takanori Sato, Yuichi Saito, Hideki Kitahara, Yoshio Kobayashi

**Affiliations:** Department of Cardiovascular Medicine, Chiba University Graduate School of Medicine, Chiba 260-8670, Japan

**Keywords:** GRACE risk score, acute coronary syndrome, percutaneous coronary intervention

## Abstract

The GRACE risk score is established to predict thrombotic events in patients with acute coronary syndrome (ACS). Although thrombotic events including myocardial infarction after ACS are mainly attributable to vulnerable plaque formation, whether the GRACE score correlates with coronary lipid-rich plaque is unclear. A total of 54 patients with ACS undergoing primary percutaneous coronary intervention under near-infrared spectroscopy intravascular ultrasound (NIRS-IVUS) guidance were included in a prospective manner. Patients were divided into two groups according to the median of the GRACE risk score. Coronary lipid plaques in the target vessel were assessed by NIRS-IVUS with lipid core burden index (LCBI) and a maximum LCBI in 4 mm (maxLCBI_4mm_). The receiver operating characteristics (ROC) curve analysis was performed based on the major adverse cardiovascular events as an exploratory analysis. The GRACE risk score was significantly and positively correlated with LCBI (*r* = 0.31, *p* = 0.03) and maxLCBI_4mm_ (*r* = 0.38, *p* = 0.006). LCBI (111.7 ± 85.7 vs. 169.0 ± 83.5, *p* = 0.02) and maxLCBI_4mm_ (428.5 ± 227.1 vs. 600.6 ± 227.7, *p* = 0.009) in the target vessel were significantly higher in the high GRACE risk score group than their counterpart. In the ROC curve analysis, LCBI and maxLCBI_4mm_ were predictive for clinical events. In conclusion, the higher GRACE risk score may serve as a discriminator of risk comprising more lipid-rich plaques as an underlying mechanism of an increased risk of thrombotic events after ACS. In patients with ACS, the higher GRACE risk score was significantly and modestly associated with greater coronary lipid plaques in the target vessel.

## 1. Introduction

The Global Registry of Acute Coronary Events (GRACE) risk score was originally established to predict short-term (in-hospital) mortality in patients with acute coronary syndrome (ACS) [1], and is currently the guideline-recommended risk model for guiding management of ACS [2,3]. Although the GRACE risk score was developed when primary percutaneous coronary intervention (PCI) was not a standard-of-care procedure [1], it is still one of the most robust risk-predicting models in a setting of ACS among numerous risk scoring systems [4,5]. We previously showed that the GRACE risk score was useful to predict short- and long-term clinical outcomes after acute myocardial infarction (MI) in the current era [6]. The current European guidelines recommend the GRACE risk score as a risk assessment tool (Class IIa), particularly in patients with non-ST segment elevation ACS [3]. In previous studies, patients with non-ST segment elevation ACS and a GRACE risk score > 140 benefited from an early invasive strategy, whereas those with a GRACE risk score ≤ 140 did not [7,8]. The higher GRACE risk score is associated with an increased risk of thrombotic events such as death and MI at mid- to long-term follow-up [9,10]. Spontaneous MI is caused by a luminal thrombus or a sudden plaque hemorrhage imposed on an atherosclerotic plaque [11]. Plaque rupture is the most frequent pathological mechanism of intracoronary thrombosis [11,12], followed by plaque erosion and calcified nodule [13]. Plaque rupture is characterized by fibroatheroma with cap disruption and luminal thrombus communicating with the underlying necrotic core, in which coronary lipid plaques play a major role [13]. In this context, we hypothesized that ACS patients with high GRACE risk score may have vulnerable coronary plaques. The identification of vulnerable patients and lesions can lead to intensive therapeutic managements including a lipid-lowering therapy, potentially resulting in a relevant prognostic benefit as a secondary prevention in patients with ACS [14]. However, only a few studies have indirectly addressed the relation between GRACE risk score and coronary atherosclerosis using angiographic scoring systems such as the SYNTAX and Gensini scores [15,16,17]. For instance, a single-center, cross-sectional study showed that among patients with ACS, the GRACE risk score was positively correlated with the SYNTAX score (*r* = 0.32, *p* < 0.001) [15], indicating that patients with the higher GRACE risk score are likely to have more complex coronary lesions, although the angiographic assessment does not directly provide information on coronary lipid plaques. Recently, near-infrared spectroscopy-intravascular ultrasound (NIRS-IVUS) has emerged as a unique intracoronary imaging modality to specifically detect lipid core plaques (LCP) and quantify coronary lipid accumulation. In prior literature, large-scale prospective studies confirmed that LCP assessed by NIRS-IVUS was significantly associated with future major adverse cardiovascular events (MACE) during short- and long-term follow-up in patient- and lesion-levels [18,19,20,21]. A recent multicenter, prospective study confirmed that the amount of LCP derived by NIRS-IVUS can predict patient-level nonculprit lesion-related MACE in patients with recent MI during median 3.7-year follow-up [21]. In the present study, we aimed to assess the relation between GRACE risk score and coronary LCP in patients with ACS.

## 2. Materials and Methods

### 2.1. Study Population

A total of 54 patients with ACS who underwent PCI under NIRS-IVUS guidance between March 2017 and December 2019 at Chiba University Hospital were prospectively enrolled (University Hospital Medical Information Network Clinical Trials Registry: UMIN000027641). The use of NIRS-IVUS was left to operator’s discretion. No exclusion criteria were applied. ACS was defined as acute MI < 48 h from the onset or unstable angina. Acute MI including both ST-segment elevation MI (STEMI) and non-ST segment elevation MI (NSTEMI) was determined according to the fourth universal definition of MI [22], defined as a rise and/or fall of cardiac troponin values with at least one value above the 99th percentile upper reference limit and with at least one of the following criteria: symptoms of acute myocardial ischemia, new ischemic electrocardiogram changes, development of pathological Q waves, imaging evidence of new loss of viable myocardium or new regional wall motion abnormality in a pattern consistent with an ischemic etiology, and identification of a coronary thrombus by angiography. Unstable angina was diagnosed using Braunwald criteria with the evidence of significant epicardial coronary artery disease on coronary angiography [23]. This study was done in accordance with the Declaration of Helsinki and was approved by the ethical committee of Chiba University Hospital, and written informed consent was obtained from all participants.

### 2.2. GRACE Risk Score

The GRACE risk score was calculated for individual participants as previously reported [1,24]. The GRACE risk score values a score for individual predictive factors (age, heart rate, systolic blood pressure, serum creatinine level, Killip class, cardiac arrest at hospital admission, elevated cardiac biomarkers, and ST-segment deviation). Heart rate, blood pressure, Killip class, creatinine level, and cardiac biomarkers were assessed at the time of admission. ST-segment deviation included ST-segment elevations or depressions of at least 1 mm in the anterior, inferior, or lateral leads on electrocardiograms. Killip class was defined as class I-absent rales and S3, class II-rales < 50% lung fields, class III-rales > 50% lung fields, and class IV-shock. Elevated cardiac biomarkers were defined as positive high-sensitivity troponin I or other cardiac enzymes (more than upper reference limit). Patients were divided into two groups based on the median of the GRACE score in this study.

### 2.3. NIRS-IVUS Imaging

Coronary angiography was performed according to standard methods [25], and PCI procedures were done per local standards with drug-eluting stent implantation [26,27,28,29,30]. After intracoronary administration of isosorbide dinitrate, the NIRS-IVUS catheter system (TVC imaging system, Infraredx, Burlington, VT, USA) was advanced beyond the culprit lesion before predilation, if possible. When the thrombolysis in myocardial infarction (TIMI) flow grade was <3 or the NIRS-IVUS catheter could not cross the target lesion, NIRS-IVUS imaging was done after predilation. Motorized pullback was performed from the distal segment to ostium of coronary artery or guiding catheter. NIRS data before coronary stent implantation were analyzed to obtain the fraction of yellow pixels on the chemogram for calculating the lipid core burden index (LCBI) in the target vessel. LCBI represents the proportion of yellow pixels (probability of lipid > 0.6) in the plaque, reported on a scale of 0 to 1000 (signifying 0% to 100% lipid). Maximal LCBI over any 4-mm segment (maxLCBI_4mm_) was also automatically calculated based on the number of yellow pixels over each possible 4-mm long axial segment in the target vessel [31,32]. Offline grayscale IVUS analysis was performed in the target lesion by using computerized planimetry (EchoPlaque 3.0, Idec Systems, Mountain View, CA, USA) according to the consensus document [33]. All NIRS-IVUS analyses were performed by experienced operators who were blinded to patients’ characteristics. MaxLCBI_4mm_ > 400 was used to define the high maxLCBI_4mm_ group in clinical outcome evaluation [20]. A culprit lesion was defined as the segment that was subsequently covered by coronary stents, and a nonculprit lesion was determined as the longer segment proximal or distal to the treated site in the target vessel.

### 2.4. Definitions

Dyslipidemia, hypertension, and diabetes were defined based on the Japanese Association of Cardiovascular Intervention and Therapeutics criteria [34]. Hypertension was defined as having a previous diagnosis of hypertension or previous antihypertensive medications, or a new diagnosis of hypertension during hospitalization with systolic blood pressure ≥ 140 mm Hg and/or diastolic blood pressure ≥ 90 mm Hg. Diabetes was defined as having a previous diagnosis of diabetes or previous glucose-lowering medications, or hemoglobin A1c ≥ 6.5% on admission. Dyslipidemia was defined as low-density lipoprotein cholesterol ≥ 140 mg/dL, high-density lipoprotein cholesterol < 40 mg/dL, or fasting triglycerides > 150 mg/dL, or a previous diagnosis of dyslipidemia. Low- and high-density lipoprotein cholesterol levels were assessed in either a fasting or nonfasting state. Other laboratory data such as hemoglobin and glomerular filtration rate was evaluated on admission. In addition, a history of smoking within the past year was defined as current smoking [34].

### 2.5. Endpoint and Statistical Analysis

The primary interest of the present study was the relation of GRACE risk score to LCBI and maxLCBI_4mm_ in the target vessel. LCBI and maxLCBI_4mm_ in the nonculprit lesion in the target vessel were also assessed. MACE, a composite of all-cause death, MI, and stroke, during the index hospitalization and after discharge were evaluated as an exploratory analysis. The admission date was set at day 0. Statistical analysis was performed by using JMP Pro 15 software (SAS Institute, Cary, NC, USA). All data are expressed as mean ± standard deviation, median [interquartile range], or frequencies and percentages, as appropriate. Continuous variables were compared with Student’s *t*-test or Mann–Whitney U-test, and categorical variables were assessed by using Fisher’s exact test. The relation between continuous variables was analyzed by using Pearson’s correlation coefficient. The receiver operating characteristics (ROC) curve analysis was performed based on the occurrence of MACE, and the best cutoff value was determined by finding the value that corresponded to the maximum average sensitivity and specificity. Because of the exploratory nature of this study, no sample size calculation was performed. A *p* value < 0.05 was considered statistically significant.

## 3. Results

Of 54 patients with ACS undergoing PCI, 15 (27.8%), 25 (46.3%), and 14 (25.9%) were presented with STEMI, NSTEMI, and unstable angina, respectively. Patients were divided into two groups with the median GRACE risk score of 117 [91.8–141.3], and their baseline characteristics are shown in Table 1. Patients in the high GRACE risk score group were older and less likely to be men and had higher rates of a history of heart failure and lower estimated glomerular filtration rate and hemoglobin levels, as compared with those in the low GRACE risk score group. Grayscale IVUS findings in the target lesion are listed in Table 2, in which no significant differences were found between patients with high and low GRACE risk scores.

**Figure 1:**
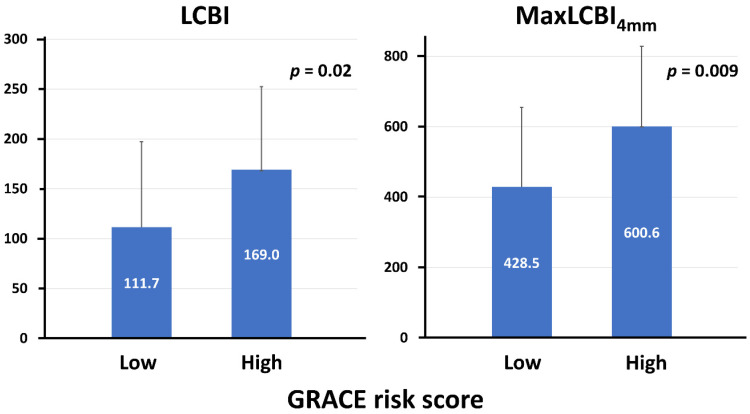
LCBI and maxLCBI_4mm_ in the target vessel in the low (<116) and high (≥116) GRACE score groups. LCBI, lipid core burden index; maxLCBI_4mm_, maximum lipid core burden index in 4 mm.

**Figure 2:**
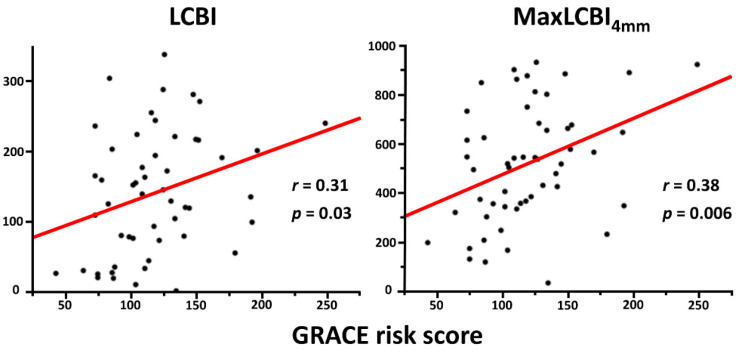
Correlation between GRACE risk score and LCBI or maxLCBI_4mm_ in the entire target vessel. LCBI, lipid core burden index; maxLCBI_4mm_, maximum lipid core burden index in 4 mm.

**Figure 3:**
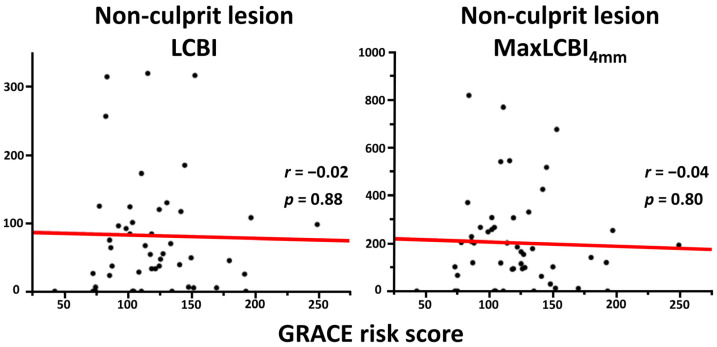
Correlation between GRACE risk score and LCBI or maxLCBI_4mm_ in the nonculprit lesion in the target vessel. LCBI, lipid core burden index; maxLCBI_4mm_, maximum lipid core burden index in 4 mm.

**Figure 4:**
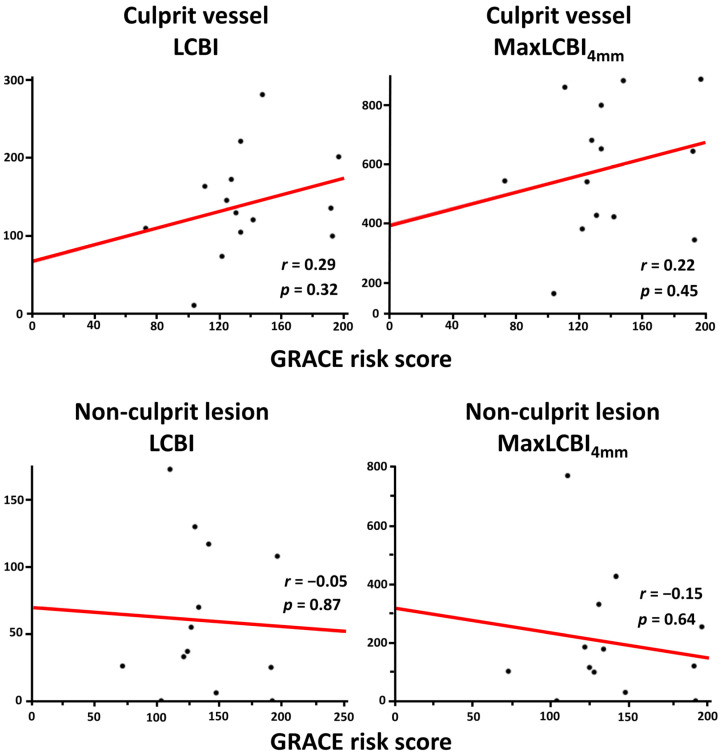
Correlation between GRACE risk score and LCBI or maxLCBI_4mm_ in patients with ST segment elevation myocardial infarction. LCBI, lipid core burden index; maxLCBI_4mm_, maximum lipid core burden index in 4 mm.

**Figure 5:**
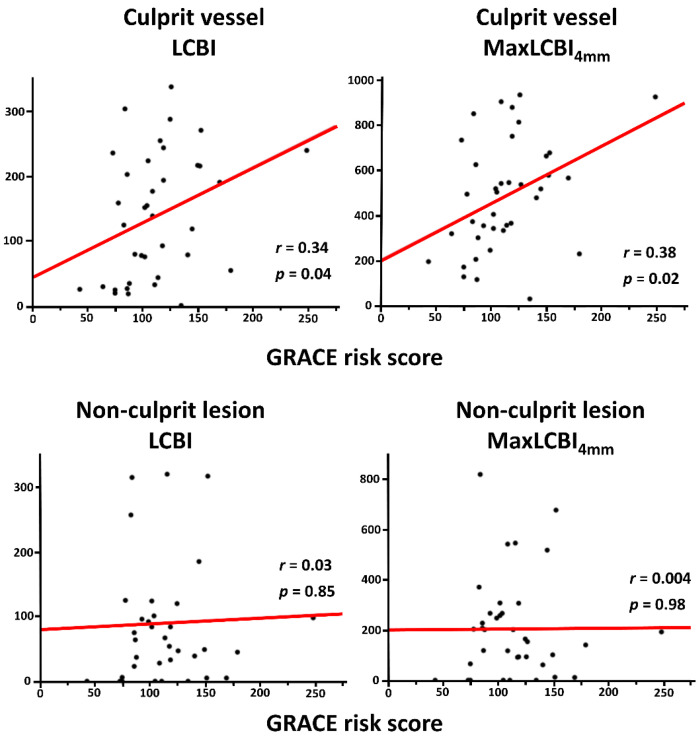
Correlation between GRACE risk score and LCBI or maxLCBI_4mm_ in patients with non-ST segment elevation myocardial infarction. LCBI, lipid core burden index; maxLCBI_4mm_, maximum lipid core burden index in 4 mm.

Overall, mean LCBI and maxLCBI_4mm_ in the target vessel were 140.0 ± 88.6 and 514.6 ± 241.3, respectively. LCBI and maxLCBI_4mm_ in the culprit lesions (205.7 ± 145.4 and 488.0 ± 263.6) were higher than those in the nonculprit lesions (82.4 ± 92.6 and 203.3 ± 200.1). Patients in the high GRACE risk score group had significantly higher LCBI and maxLCBI_4mm_ in the target vessel than their counterpart (Figure 1). The high GRACE risk score was significantly associated with a higher likelihood of having maxLCBI_4mm_ > 400 (77.8% vs. 44.4%, *p* = 0.01). In addition, there was a significant positive correlation between the GRACE risk score and LCBI or maxLCBI_4mm_ in the entire target vessel (Figure 2), although this trend was not seen in the nonculprit lesion in the target vessel (Figure 3). When focusing on LCBI and maxLCBI_4mm_ in the target vessel and in the nonculprit lesion in patients with either STEMI or non-ST segment elevation ACS, the overall results were similar to those in the entire study population (Figure 4 and Figure 5).

**Figure 6:**
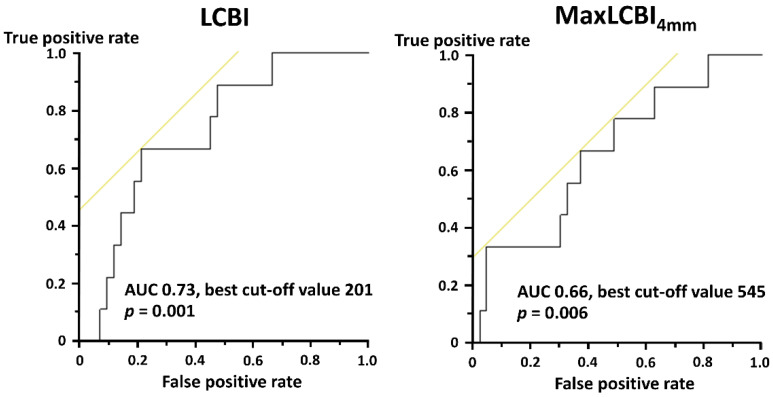
Receiver operating characteristics curve for major cardiovascular events AUC, area under the curve. LCBI, lipid core burden index; maxLCBI_4mm_, maximum lipid core burden index in 4 mm.

During the median follow-up period of 498.5 [356.5, 1061.5] days, nine (16.7%) patients experienced major cardiovascular events. In the ROC curve analyses, LCBI (area under the curve 0.73, best cut-off value 201, sensitivity 66.7%, specificity 78.6%, *p* = 0.001) and maxLCBI_4mm_ (area under the curve 0.66, best cut-off value 545, sensitivity 66.7%, specificity 62.8%, *p* = 0.006) were significantly associated with the occurrence of MACE (Figure 6). The rates of clinical events in the high and low GRACE risk score groups with and without maxLCBI_4mm_ > 400 are shown in Table 3. Despite the lack of statistical significance, patients with the high GRACE score and maxLCBI_4mm_ > 400 were likely to develop MACE (Table 3). Table 4 shows that patients with high GRACE risk score and high maxLCBI_4mm_ with the best cutoff value of 545 had a significantly higher risk of MACE, and the results were similar in combinations of GRACE score and LCBI with the cutoff value (Table 5). All recurrent MI were attributable to events in the nonculprit vessel.

## 4. Discussion

The present study demonstrated that the GRACE risk score was positively correlated with LCBI and maxLCBI_4mm_ and that LCBI and maxLCBI_4mm_ in the target vessel were significantly higher in the high GRACE risk score group than their counterpart. In addition, LCBI and maxLCBI_4mm_ in the target vessel was significantly predictive for MACE, and patients with the high GRACE score and high LCBI and maxLCBI_4mm_ were at an increased risk of subsequent ischemic events.

### 4.1. Relation between GRACE Risk Score and Lipid Plaques

In the original GRACE study, the higher risk score was associated with an increased risk of death or MI [9]. In a previous report, the rates of death or MI at one year in patients with non-ST segment elevation ACS were progressively increased with the increase in the GRACE score categories (4.2% vs. 9.6% vs. 11.9% vs. 27.3% in the GRACE risk score < 96, 96–112, 113–133, and >133 categories) [35]. Thus, ACS patients with high GRACE risk score may be likely to have vulnerable coronary plaques given the high rates of ischemic events including death and MI in the previous studies [9,35], and the fact that coronary plaque rupture of LCP is the most frequent cause of spontaneous MI, which is defined as disruption of the accumulation of acellular, lipid-rich material in the intima [11,12]. Previous reports showed that the higher GRACE risk score was associated with the higher coronary anatomical complexity using angiographic scoring systems (e.g., SYNTAX and Gensini scores) [15,16,17]. For instance, Cakar et al. showed that among patients with non-ST segment elevation ACS, the higher GRACE risk score was associated with a higher Gensini score and a higher likelihood of having multivessel disease [17]. Although these angiographic scores do not directly represent coronary plaque vulnerability, NIRS-IVUS, a novel intracoronary imaging system, can uniquely quantify and detect LCP in vivo [32,36,37]. A large-scale prospective cohort study (*n* = 1563) demonstrated that LCBI and maxLCBI_4mm_ predicted MACE, especially when a patient had maxLCBI_4mm_ > 400 (adjusted hazard ratio 1.89, 95% confidence interval 1.26–2.83, *p* = 0.002) [20]. More recently, a multicenter, prospective study confirmed that maxLCBI_4mm_ > 400 was an independent predictor of patient-level nonculprit lesion-related MACE (adjusted odds ratio 2.27, 95% confidence interval 1.25–4.13) in patients with recent MI during median 3.7 years follow-up [21]. Thus, NIRS-IVUS can be useful for detecting vulnerable plaques and high-risk patients and predicting future MACE. In this context, the present study showed that the higher GRACE risk score was associated with the higher LCBI and maxLCBI_4mm_. Given that more than three-quarter of patients with the GRACE risk score ≥ 117 had maxLCBI_4mm_ > 400 in the present study, ACS patients with high GRACE score may have not only high-risk patient profiles but also high-risk (lipid-rich) coronary lesions.

### 4.2. GRACE Risk Score Guided Management

While NIRS-IVUS guided PCI is not always feasible, the GRACE risk score is easy to calculate on admission. Therefore, the GRACE risk score may be useful to stratify a risk of future MI in patients with ACS, potentially translating into modification of secondary preventive strategies. For instance, an ACS patient with very high GRACE risk score is likely to have vulnerable coronary plaques (e.g., maxLCBI_4mm_ > 400), and thus the patient would benefit from intensified lipid-lowering therapies. Several reports have demonstrated that aggressive low-density lipoprotein cholesterol lowering by using proprotein convertase subtilisin/kexin type 9 (PCSK9) inhibitors was associated with reduced plaque volumes and LCBI and maxLCBI_4mm_ on NIRS-IVUS [38,39]. The recently reported PACMAN-AMI and HUYGENS trials reinforced that among patients with acute MI, the addition of subcutaneous PCSK9 inhibitor, relative to placebo, to statin therapy resulted in significantly greater coronary plaque regression and fibrous cap thickness in non-infarct-related arteries at one year [40,41]. Given that PCSK9 inhibitors was reported to maintain levels of adherence and quality of life [42,43], this treatment strategy may have a potential to improve outcomes in high-risk ACS patients. In addition, as a secondary prevention of ACS, antiplatelet therapy after PCI is also essential [44]. Despite an increased risk of bleeding, potent and prolonged antiplatelet therapy may be beneficial in selected high-risk patients with ACS [44]. Thus, some patients with ACS who have high GRACE risk score with potential vulnerable plaques may be a candidate for intensive medical treatment such as PCSK9 inhibition and potent antiplatelet therapy. The impact of GRACE risk score-guided decision-making on invasive strategies is being tested in large-scale randomized trials [45,46], but further studies are also needed to determine whether GRACE risk score-guided preventive strategies are feasible and effective in clinical practice.

### 4.3. Study Limitations

Some limitations to our study should be acknowledged. This was a single-center study, and the analysis was performed in a post-hoc manner despite the prospective design. Because of the small sample size and modest correlations of GRACE risk score with LCBI and maxLCBI_4mm_, the present study results should be considered hypothesis-generating. Multivariable analysis seemed useful to determine the effect of GRACE risk score on coronary plaque vulnerability beyond age, chronic kidney disease, and anemia, all of which are a surrogate of frailty and were significantly more prevalent in patients with high GRACE score (Table 1). However, the small sample size precluded multivariable adjustment and the analysis on incremental prognostic values of LCBI and maxLCBI_4mm_ compared to the GRACE risk score alone. Because NIRS-IVUS system has a relatively large profile and its use was left to operators’ discretion, the present study population may represent a low-risk population without severe calcification and tortuosity, namely a selection bias [47]. Although NIRS-IVUS was used in all patients, the impact of other modalities evaluating coronary plaque morphology (e.g., optical coherence tomography) is unknown [48,49]. In this study, the significant relation of GRACE risk score to LCBI or maxLCBI_4mm_ were observed in the entire culprit vessel, whereas the relationship was unclear in the nonculprit lesion. Given that the prognostic ability of NIRS-IVUS with LCBI or maxLCBI_4mm_ has been extensively investigated in previous studies in those in nonculprit segments [20,21], the present study results should be interpreted with caution. Although the large-scale subanalysis of COLOR registry showed that pre-PCI LCBI in the culprit lesion was not significantly associated with subsequent culprit lesion-related events after PCI, the relation of culprit lesion LCBI to nonculprit lesion related events and other clinical outcomes remains unclear [50]. We believe that future studies addressing the prognostic impact of LCBI and maxLCBI_4mm_ in the culprit lesion and the relation of GRACE risk score to coronary plaque vulnerability in the nonculprit segment are warranted.

## 5. Conclusions

In patients with ACS, a higher GRACE risk score was significantly associated with greater coronary lipid plaques in the target vessel. The presence of vulnerable plaques assessed with NIRS-IVUS might convey prognostic impact in patients with acute MI in combination with GRACE risk score. Future studies are needed to determine whether the GRACE risk score-guided preventive strategies are clinically effective in patients with ACS.

## Figures and Tables

**Table 1:** Baseline characteristics of patients with high and low GRACE risk scores.

Variable	All (*n* = 54)	High (*n* = 27)	Low (*n* = 27)	*p* Value
Age (years)	70.1 ± 10.4	76.6 ± 7.4	63.7 ± 9.1	<0.001
Men	41 (75.9%)	17 (63.0%)	24 (88.9%)	0.02
Body mass index (kg/m^2^)	23.6 ± 3.3	23.0 ± 3.3	24.2 ± 3.3	0.19
Hypertension	36 (66.7%)	19 (70.4%)	17 (63.0%)	0.56
Diabetes mellitus	20 (37.0%)	11 (40.7%)	9 (33.3%)	0.57
Dyslipidemia	39 (72.2%)	17 (63.0%)	22 (81.5%)	0.13
Current smoker	12 (22.2%)	8 (29.6%)	4 (14.8%)	0.19
Prior MI	10 (18.5%)	5 (18.5%)	5 (18.5%)	1.00
Prior PCI	15 (27.8%)	5 (18.5%)	10 (37.0%)	0.13
Prior CABG	2 (3.7%)	1 (3.7%)	1 (3.7%)	1.00
Prior heart failure	5 (9.3%)	5 (18.5%)	0 (0%)	0.006
Hemodialysis	1 (5.9%)	1 (11.1%)	0 (0%)	0.25
eGFR (mL/min/1.73 m^2^)	70.0 ± 22.0	58.1 ± 19.7	81.1 ± 18.2	<0.001
Hemoglobin (g/dL)	13.6 ± 2.1	13.0 ± 2.4	14.1 ± 1.5	0.04
Total cholesterol (mg/dL)	183.8 ± 35.9	184.2 ± 33.9	183.5 ± 38.7	0.94
HDL cholesterol (mg/dL)	53.4 ± 18.9	52.2 ± 17.3	54.6 ± 20.7	0.64
LDL cholesterol (mg/dL)	107.6 ± 30.9	111.7 ± 34.2	103.4 ± 27.1	0.34
Triglyceride (mg/dL)	149.5 ± 116.3	133.1 ± 96.5	165.9 ± 133.1	0.30
Hemoglobin A1c (%)	6.2 ± 0.9	6.4 ± 1.1	6.1 ± 0.6	0.24
Killip class on admission				<0.001
I	43 (79.6%)	16 (59.3%)	27 (100%)	
II	4 (7.4%)	4 (14.8%)	0 (0%)	
III	1 (1.9%)	1 (3.7%)	0 (0%)	
IV	6 (11.1%)	6 (22.2%)	0 (0%)	
Clinical presentation				<0.001
STEMI	15 (27.8%)	12 (44.4%)	3 (11.1%)	
NSTEMI	25 (46.3%)	14 (51.9%)	11 (40.7%)	
Unstable angina	14 (25.9%)	1 (3.7%)	13 (48.2%)	
GRACE risk score	121.2 ± 39.8	150.7 ± 33.3	91.7 ± 17.7	<0.001
Medications				
β-blocker	18 (33.3%)	10 (37.0%)	8 (29.6%)	0.56
ACE-I or ARB	34 (63.0%)	19 (70.4%)	15 (55.6%)	0.26
Calcium channel blocker	17 (31.5%)	6 (22.2%)	11 (40.7%)	0.14
Diuretic	9 (1.7%)	22 (81.5%)	20 (74.1%)	0.51
Statin	42 (77.8%)	8 (29.6%)	1 (3.7%)	0.007
Culprit vessel				0.68
RCA	18 (33.3%)	9 (33.3%)	9 (33.3%)	
LMT/LAD	29 (53.7%)	14 (51.9%)	15 (55.6%)	
LCX	7 (13.0%)	4 (14.8%)	3 (11.1%)	
Three vessel disease	11 (20.4%)	4 (14.8%)	7 (25.9%)	0.31
Final TIMI flow grade				0.55
≤2	3 (7.4%)	2 (7.4%)	1 (3.7%)	
3	51 (94.4%)	25 (92.6%)	26 (96.3%)	

High and low GRACE risk score groups were divided with the median value of 117. ACE-I, angiotensin converting enzyme inhibitor; ARB, angiotensin receptor blocker; CABG, coronary artery bypass grafting; eGFR, estimated glomerular filtration rate; HDL, high-density lipoprotein; LAD, left anterior descending artery; LCX, left circumflex; LDL, low-density lipoprotein; LMT, left main trunk; MI, myocardial infarction; NSTEMI, non-ST segment elevation myocardial infarction; PCI, percutaneous coronary intervention; RCA, right coronary artery; STEMI, ST segment elevation myocardial infarction; TIMI, thrombolysis in myocardial infarction.

**Table 2:** Grayscale IVUS findings between patients with high and low GRACE risk scores.

Variable	All (*n* = 54)	High (*n* = 27)	Low (*n* = 27)	*p* Value
Length (mm)	33.4 ± 16.3	34.3 ± 18.9	32.8 ± 13.9	0.76
Lumen area (mm^2^)	4.6 ± 3.4	3.6 ± 1.4	5.6 ± 4.4	0.06
Vessel area (mm^2^)	17.4 ± 6.9	15.7 ± 5.1	19.0 ± 8.0	0.13
Plaque area (mm^2^)	12.7 ± 6.0	12.1 ± 4.9	13.4 ± 7.0	0.51
Plaque burden (%)	72.6 ± 13.9	75.6 ± 10.5	69.5 ± 16.3	0.17

**Table 3:** Clinical outcomes with the cut-off value of maxLCBI_4mm_ of 400.

Variable	All (*n* = 54)	High GRACE		Low GRACE		*p* Value
		High maxLCBI_4mm_	Low maxLCBI_4mm_	High maxLCBI_4mm_	Low maxLCBI_4mm_	
		(*n* = 21)	(*n* = 6)	(*n* = 12)	(*n* = 15)	
MACE	9 (16.7%)	5 (23.8%)	0 (0%)	2 (16.7%)	2 (13.3%)	0.39
All-cause death	6 (11.1%)	5 (23.8%)	0 (0%)	0 (0%)	1 (6.7%)	0.06
Recurrent MI	4 (7.4%)	1 (4.8%)	0 (0%)	2 (16.7%)	1 (6.7%)	0.51
Stroke	1 (2.0%)	0 (0%)	0 (0%)	0 (0%)	1 (6.7%)	0.48

High and low GRACE risk score groups were divided with the median value of 117. High and low maxLCBI_4mm_ groups were defined as > and ≤400. MACE, major adverse cardiovascular events; maxLCBI_4mm_, maximal lipid core burden index over any 4-mm segment; MI, myocardial infarction.

**Table 4:** Clinical outcomes with the cut-off value of maxLCBI_4mm_ of 545.

Variable	All (*n* = 54)	High GRACE		Low GRACE		*p* Value
		High maxLCBI_4mm_	Low maxLCBI_4mm_	High maxLCBI_4mm_	Low maxLCBI_4mm_	
		(*n* = 15)	(*n* = 12)	(*n* = 6)	(*n* = 21)	
MACE	9 (16.7%)	5 (33.3%)	0 (0%)	0 (0%)	4 (19.1%)	0.03
All-cause death	6 (11.1%)	5 (33.3%)	0 (0%)	0 (0%)	1 (4.8%)	0.01
Recurrent MI	4 (7.4%)	0 (0%)	1 (8.3%)	0 (0%)	3 (14.3%)	0.22
Stroke	1 (1.9%)	0 (0%)	0 (0%)	0 (0%)	1 (4.8%)	0.59

High and low GRACE risk score groups were divided with the median value of 117. High and low maxLCBI_4mm_ groups were defined with the best cutoff value of 545. MACE, major adverse cardiovascular events; maxLCBI_4mm_, maximal lipid core burden index over any 4 mm segment; MI, myocardial infarction.

**Table 5:** Clinical outcomes with the cut-off value of LCBI of 201.

Variable	All (*n* = 54)	High GRACE		Low GRACE		*p* Value
		High LCBI	Low LCBI	High LCBI	Low LCBI	
		(*n* = 9)	(*n* = 18)	(*n* = 5)	(*n* = 22)	
MACE	9 (16.7%)	3 (33.3%)	2 (11.1%)	2 (40.0%)	2 (9.1%)	0.21
All-cause death	6 (11.1%)	3 (33.3%)	2 (11.1%)	0 (0%)	1 (4.6%)	0.14
Recurrent MI	4 (7.4%)	0 (0%)	1 (5.6%)	2 (40.0%)	1 (4.6%)	0.12
Stroke	1 (1.9%)	0 (0%)	0 (0%)	0 (0%)	1 (4.6%)	0.61

High and low GRACE risk score groups were divided with the median value of 117. High and low LCBI groups were defined as > and ≤201. MACE, major adverse cardiovascular events; maxLCBI_4mm_, maximal lipid core burden index over any 4 mm segment; MI, myocardial infarction.

## Data Availability

The data presented in this study are available on reasonable request.
